# Identification of leaf rust resistance genes *Lr34* and *Lr46* in common wheat (*Triticum aestivum* L. ssp. *aestivum*) lines of different origin using multiplex PCR

**DOI:** 10.1515/biol-2021-0018

**Published:** 2021-02-20

**Authors:** Agnieszka Tomkowiak, Roksana Skowrońska, Michał Kwiatek, Julia Spychała, Dorota Weigt, Danuta Kurasiak-Popowska, Janetta Niemann, Sylwia Mikołajczyk, Jerzy Nawracała, Przemysław Łukasz Kowalczewski, Kinza Khan

**Affiliations:** Department of Genetics and Plant Breeding, Poznań University of Life Sciences, 11 Dojazd St., Poznań, Poland; Department of Food Technology of Plant Origin, Poznań University of Life Sciences, 31 Wojska Polskiego St., 60-624, Poznań, Poland

**Keywords:** common wheat, leaf rust, multiplex PCR, *Puccinia recondita* f. sp. *tritici*, resistance genes, SSR molecular markers

## Abstract

Leaf rust caused by the fungus *Puccinia recondita* f. sp. *tritici* is one of the most dangerous diseases of common wheat. Infections caused by fungal pathogens reduce the quantity and quality of yields of many cereal species. The most effective method to limit plant infection is to use cultivars that show rust resistance. Genetically conditioned horizontal-type resistance (racial-nonspecific) is a desirable trait because it is characterized by more stable expression compared to major (R) genes that induce racially specific resistance, often overcome by pathogens. Horizontal resistance is conditioned by the presence of slow rust genes, which include genes *Lr34* and *Lr46*. This study aimed to identify markers linked to both genes in 64 common wheat lines and to develop multiplex PCR reaction conditions that were applied to identify both genes simultaneously. The degree of infestation of the analyzed lines was also assessed in field conditions during the growing season of 2017 and 2018. Simple sequence repeat anchored-polymerase chain reaction (SSR-PCR) marker *csLV* was identified during analysis in line PHR 4947. The presence of a specific sequence has also been confirmed in multiplex PCR analyses. In addition to gene *Lr34*, gene *Lr46* was identified in this genotype. Lines PHR 4947 and PHR 4819 were characterized by the highest leaf rust resistance in field conditions. During STS-PCR analyses, the marker *wmc44* of gene *Lr46* was identified in most of the analyzed lines. This marker was not present in the following genotypes: PHR 4670, PHR 4800, PHR 4859, PHR 4907, PHR 4922, PHR 4949, PHR 4957, PHR 4995, and PHR 4997. The presence of a specific sequence has also been confirmed in multiplex PCR analyses. Genotypes carrying the markers of the analyzed gene showed good resistance to leaf rust in field conditions in both 2017 and 2018. Research has demonstrated that marker assisted selection (MAS) and multiplex PCR techniques are excellent tools for selecting genotypes resistant to leaf rust.

## 1 Introduction

Common wheat (*Triticum aestivum* L. ssp. *aestivum*) is a crop that occupies a leading place in global food production [1]. The presence of many cultivars allows us to grow wheat in most climate zones, which results in its largest proportion among cultivated cereals in the world. Because of the dynamic development of wheat cultivation and production, diseases of seed plantations are increasingly being observed. Fungal diseases pose a particular threat, as they limit the leaf assimilation area and cause a significant yield loss [2,3]. Wheat leaf rust caused by *Puccinia recondita* f. sp. *tritici* is one of the most dangerous and common fungal diseases. Currently, the most beneficial method of plant protection is conducting resistance breeding, consisting in the identification and selection of resistant cultivars and transferring resistance genes to other cultivars for their pyramidization.

In hexaploid wheat, leaf rust resistance genes are widespread in the genome. Different genes determine resistance to emerging types of infection [4]. An essential direction in resistance breeding is the search for new and effective sources of resistance to wheat diseases [5]. The number of genes that determine avirulence and their expression vary between *Puccinia recondita* f. sp. *tritici* populations [4]. Systematic appearance of new pathotypes is a significant problem in resistance breeding. Fungi of the genus *Puccinia* are characterized by high genetic variability and show adaptability to climatic conditions. Therefore, it is necessary to constantly search and select new sources of resistance to create new, resistant wheat cultivars [6].

The most numerous group of genes responsible for leaf rust resistance are the *Lr* (leaf rust) genes. A small group of leaf rust resistance genes is referred to as *slow rust* genes, and it includes *Lr34* [7] and *Lr46* [8]. They are characterized by persistent and nonspecific adult plant resistance (APR), while their expression effect is more limited compared to that of racially specific genes. Inheritance of these genes is quantitative, and literature data very often indicate specific quantitative trait locus (QTL) regions associated with them, which are often dispersed throughout the whole wheat genome. Horizontal resistance is a very desirable trait because it provides a longer and more stable response than the major genes, whose racial-specific resistance is often overcome by the pathogen. Literature data shows that for slow rust resistance there are three gene complexes of major importance: *Lr34*/*Yr18*/*Pm38*, *Lr46*/*Yr29*/*Pm38,* and *Sr2*/*Yr30* [9,10].

One of the well-characterized racially nonspecific resistance genes is *Lr34* [11], found on wheat chromosome 7DS. This gene determines partial resistance in adult plants to all known of *P. recondita* f. sp. *tritici*. Analyzing the effect of this gene, it was found that genotypes carrying *Lr34* also had increased resistance to stripe rust [12], stem rust [10,13], and powdery mildew [14]. These genes (also located at the locus of chromosome 7DS) have been designated as *Yr18*, *Sr57*, and *Pm38*, respectively [11]. Gene *Lr34*, previously referred to as *LrT2* [13], is present in older South American (Frontana) cultivars and derivatives of this variety. It is also found in cultivars of Chinese origin [10]. An important feature of this gene is that the resistance conditioned by *Lr34* has not been overcome in many regions, which has contributed to the stability of wheat cultivars that carry the above gene complex [11].

The leaf rust resistance gene *Lr46* is also a slow rust-type gene. Like *Lr34*, it does not provide the host plant with complete resistance to all races of *Puccinia recondita* f. sp. *tritici*, while the effect of its expression can also delay the infection process in adult plants or slow down the development of disease caused by the pathogen (https://maswheat.ucdavis.edu). As with *Lr34*, the *Lr46/Yr29/Sr58/Pm39* gene complex is located at a locus on chromosome 1BL. It has been characterized in wheat derived from CIMMYT as well as in lines originating from South American wheat cultivars [11]. Research on the effect of the *Lr46/Yr29/Sr58/Pm39* gene complex on multifactorial resistance to leaf rust, stripe rust, stem rust, and powdery mildew was described by William et al. [15]. The presence of both genes significantly extends the latency period of the disease and reduces its severity relative to the susceptible media.

## 2 Materials and methods

### 2.1 Plant materials

The plant materials used in the study were 64 lines of common wheat (*Triticum aestivum* L. ssp. *aestivum*) of various origins from different countries of Europe. The plant material comes from the common wheat collection at the experimental station of Poznańska Hodowla Roślin sp. z o.o. in Poland. According to the literature sources, some of these materials have good leaf rust resistance and may contain *Lr34* and *Lr46* resistance genes. Frontana was the control variety used in the experiment that carried both analyzed genes. The control variety Myna was also used in multiplex PCR analyses. Both cultivars were derived from the National Small Grain Collection in Agriculture Research Station Aberdeen, USA.

### 2.2 Field experiment

Field experiment with the tested wheat genotypes was conducted on experimental plots belonging to Poznańska Hodowla Roślin Sp. z o.o. (N: 51°30′44″, E: 17°50′51″) in the years 2017–2018. In the spring, along with the beginning of the vegetation period, close observations were carried out on wheat plots to find clusters of leaf rust urediniospores. Depending on the size of the leaves, 100–150 wheat leaves were harvested randomly per plot. Field evaluation of wheat was carried out in accordance with the recommendations of the European and Mediterranean Plant Protection Organization using a 9° scale. Observations were carried out on the basis of the total number of leaves in the sample, number of infected leaves (i.e., number of leaves with urediniospore clusters), percentage of infected leaves, total number of urediniospore clusters, and average number of urediniospore per wheat leaf. Wheat leaf analyses for the presence of urediniospore clusters were repeated regularly every few days until the end of the vegetation period.

Favorable conditions for plant growth and development were recorded during the 2017 growing season in the area where the field experiment was established: N: 51°30′44″, E: 17°50′51″. In March, the average temperature was 4°C. In April, despite spring frosts, the temperature exceeding 7°C also did not damage the crops. The second half of May brought a higher temperature, as a result of which vegetation accelerated significantly and low rainfall delayed the development of fungal diseases, which began to develop in the second half of June. The growing season of 2018 in this area was extremely warm compared to the entire five decades of 1966–2015. The average temperature of the growing season (from April to September) was higher than the norm (1966–2015) by about +3°C. A small amount of rainfall in March and April significantly weakened the plants, and fungal diseases appeared on them in May.

### 2.3 DNA isolation

DNA isolation of all tested common wheat samples was performed using the Plant & Fungi DNA Purification Kit (EURx), according to the recommendations attached to the protocol. Concentration and purity of the resulting preparation were measured using a spectrophotometer (DeNovix). The samples were subsequently diluted with the supplied elution buffer to obtain a uniform concentration of 70 ng/µL.

### 2.4 Genes identification

PCR-SSR amplification was carried out using a B3 Tet thermocycler (Biometra) under appropriate conditions for the analyzed genes *Lr34* and *Lr46*. The PCR reaction was performed in a 20 µL volume of the mixture per sample, where 1 µL of the mixture constituted previously isolated DNA. Mixtures differed in the volume of individual components depending on the gene tested [16].

Identification of individual genes was performed using primers with sequences derived from the MASWheat database (https://maswheat.ucdavis.edu), as listed in Table 1. The 20 µL reaction mixture for gene *Lr34* contained the following: 5× Green GoTaq^®^ Flexi Buffer – 4 µL, MgCl_2_ (2 mM) – 1.6 µL, dNTP Mix (0.2 mM each) – 0.45 µL, GoTaq® DNA Polymerase (1.25 µ) – 0.2 µL, water – 11.75 µL, primers (0.25 µM) – 2 × 0.5 µL, and DNA (50 ng/µ) – 1 µL. The 20 µL reaction mixture for gene *Lr46* consisted of the following: 5× Green GoTaq® Flexi Buffer – 4 µL, MgCl_2_ (2 mM)  – 1.8 µL dNTP Mix (0.2 mM each) – 0.5 µL, GoTaq® DNA Polymerase (1.25 µ) – 0.25 µL, water – 11.05 µL, primers (0.25 µM) – 2 × 0.7 µL, and DNA template (50 ng/µ) – 1 µL. The PCR reaction profile for both genes differed in primer annealing temperature, determined on the basis of their melting temperature, and was as follows: initial denaturation – 5 min at 94°C, then 45 cycles (denaturation – 45 s at 94°C, annealing – 30 s at 55°C (*Lr34*) or 59°C (*Lr46*), synthesis – 1 min at 72°C), final synthesis – 7 min at 72°C, and storage at 4°C for a maximum of 24 h [16].

**Table 1:** Primer sequences and their annealing temperatures for the identification of *Lr34* and *Lr46* genes

No.	Gene – primer	Primer sequence	Annealing temperature
1	*Lr34 – csLV34F*	5′-GTTGGTTAAGACTGGTGATGG-3′	
2	*Lr34 – csLV34R*	5′-TGCTTGCTATTGCTGAATAGT-3′	55°C
3	*Lr46 – wmc44F*	5′-GGTCTTCTGGGCTTTGATCCT-3′	
4	*Lr46 – wmc44R*	5′-GTTGCTAGGGACCCGTAGTGG-3′	59°C

The resulting PCR products were subjected to electrophoretic separation on a 2.5% agarose gel, using 2 µL of Midori Green Stain dye. O’RangeRuler™ 100 bp DNA Ladder was used as the molecular mass size standard. Electrophoretic separation was carried out in TBE 1× buffer for 2.5 h at 70 V. Then, to visualize PCR products, a Gel Doc™ XR UV Molecular Imager transilluminator was used together with ImageLab™ Software (Bio-Rad, USA).

In the next stage, multiplex PCR conditions were developed for *Lr34* and *Lr46* gene markers. The 20 µL PCR multiplex reaction mixture consisted of the following: 5× Green GoTaq® Flexi Buffer – 4 µL, MgCl_2_ – 1.8 µL, dNTP Mix – 0.5 µL, GoTaq® DNA Polymerase – 0.3 µL, water – 10.4 µL, primers *csLV34F* i *csLV34R* – 2 × 0.6 µL, primers *WMC44-F* i *WMC44-R* – 2 × 0.4 µL, and DNA template – 1 µL. Amplification of the multiplex PCR reaction products was carried out in the following temperature profile: initial denaturation – 5 min at 94°C, then 45 cycles (denaturation – 45 s at 94°C, primer annealing – 30 s at 57°C, and the synthesis – 1 min at 72°C), final synthesis – 7 min at 72°C, and storage at 4°C for a maximum of 24 h.

## 3 Results

The *Lr34* allele yields a 150 bp product, and a 229 bp band is amplified in non-*Lr34* germplasm. For gene *Lr46*, a 242 bp product was identified in lines carrying this gene.

### 3.1 Identification of the gene *Lr34* marker *csLV34*

**Figure A1:**
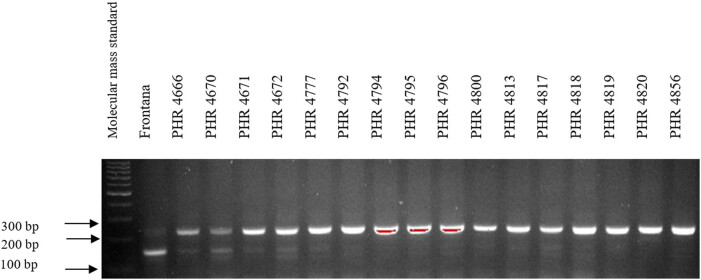
Electropherogram showing the identification of gene *Lr34* marker *csLV34* in wheat lines of different origin (genotypes 1–16).

**Figure A2:**
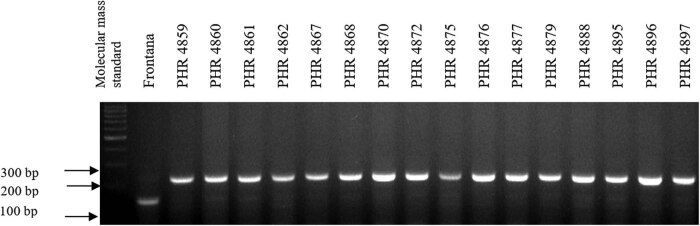
Electropherogram showing the identification of gene *Lr34* marker *csLV34* in wheat lines of different origin (genotypes 17–32).

**Figure A3:**
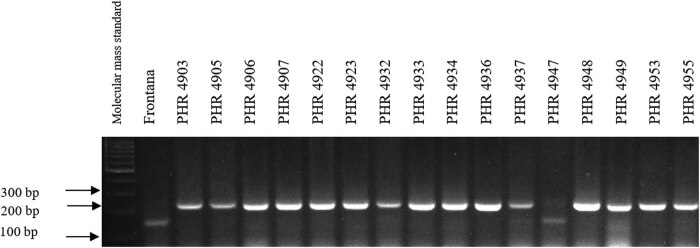
Electropherogram showing the identification of gene *Lr34* marker *csLV34* in wheat lines of different origin (genotypes 33–48).

**Figure A4:**
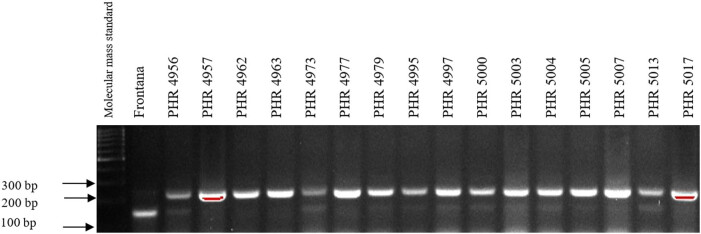
Electropherogram showing the identification of gene *Lr34* marker *csLV34* in wheat lines of different origin (genotypes 49–64).

Of the 64 lines tested, the *Lr34* marker was only present in genotype PHR 4947 (Figure A3) where a 150 bp product appeared, and in the control variety Frontana. For other lines, a 229 bp product was identified as evidence of gene absence (Figures A1–A4).

### 3.2 Identification of the gene *Lr46* marker *wmc44*

**Figure A5:**
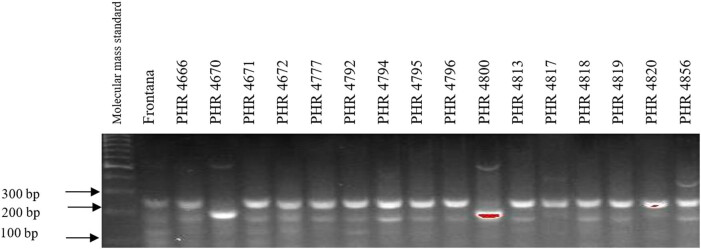
Electropherogram showing the identification of gene *Lr46* marker in wheat lines of different origin (genotypes 1–17).

**Figure A6:**
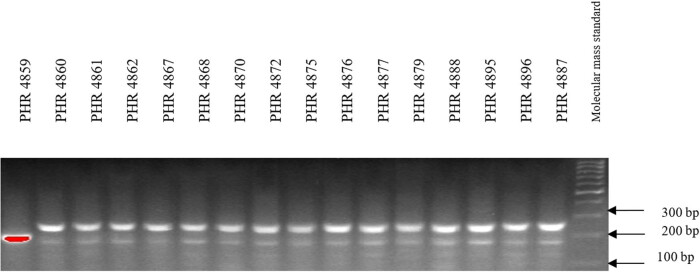
Electropherogram showing the identification of gene *Lr46* marker in wheat lines of different origin (genotypes 18–32).

**Figure A7:**
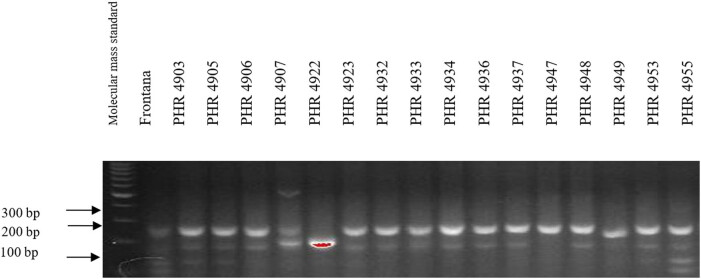
Electropherogram showing the identification of gene *Lr46* marker in wheat lines of different origin (genotypes 33–48).

**Figure A8:**
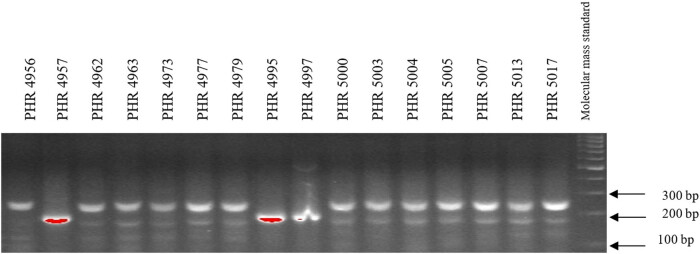
Electropherogram showing the identification of gene *Lr46* marker in wheat lines of different origin (genotypes 49–64).

Of the 64 lines tested, the gene *Lr46* marker was present in most of the genotypes, as evidenced by the presence of a 242 bp product (Figures A5–A8). The marker *wmc44* was not observed in the following lines: PHR 4670, PHR 4800, PHR 4859, PHR 4907, PHR 4922, PHR 4949, PHR 4957, PHR 4995, and PHR 4997. Nonspecific products were also observed in most lines.

### 3.3 Simultaneous identification of the gene *Lr46* marker *wmc44* and gene *Lr34* marker *csLV34* (multiplex PCR)

**Figure A9:**
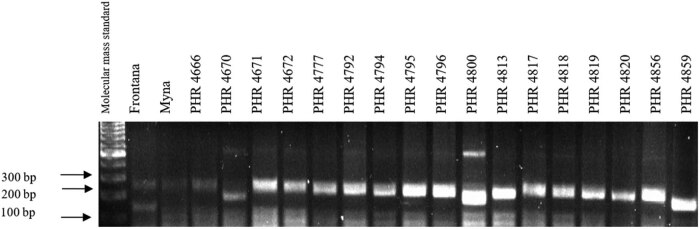
Electropherogram showing simultaneous identification of *wmc44* and *csLV* markers of *Lr34* and *Lr36* genes in wheat lines of different origin (genotypes 1–17).

**Figure A10:**
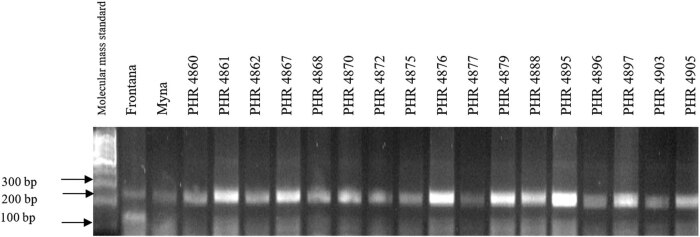
Electropherogram showing simultaneous identification of *wmc44* and *csLV* markers of *Lr34* and *Lr36* genes in wheat lines of different origin (genotypes 18–34).

**Figure A11:**
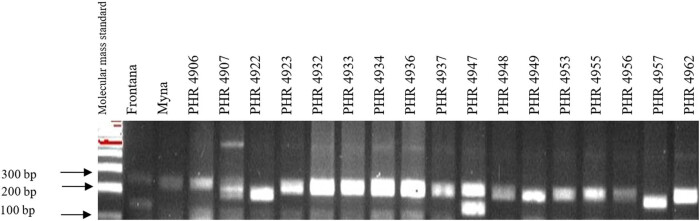
Electropherogram showing simultaneous identification of *wmc44* and *csLV* markers of *Lr34* and *Lr36* genes in wheat lines of different origin (genotypes 35–51).

**Figure A12:**
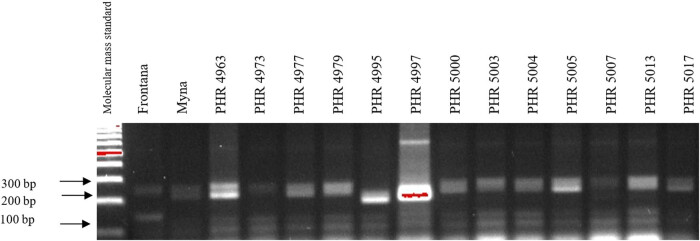
Electropherogram showing simultaneous identification of *wmc44* and *csLV* markers of *Lr34* and *Lr36* genes in wheat lines of different origin (genotypes 52–64).

Comparing the results of multiplex PCR analyses with single PCR, the result was reproducible for gene *Lr34*, i.e., the *csLV* marker of gene *Lr34* was only present in line PHR 4947 (Figure A3) where a 150 bp product appeared, and in the control variety Frontana. For gene *Lr46*, single gene and multiplex PCR analysis results were also reproducible. A 242 bp product characteristic of gene *Lr46* was not observed in the genotypes: PHR 4670, PHR 4800, PHR 4859, PHR 4907, PHR 4922, PHR 4949, PHR 4957, PHR 4995, and PHR 4997 (Figures A9–A12).

### 3.4 Observations of infection degree of the analyzed wheat genotypes in 2017–2018

In the field experiment, the highest degree of resistance to infection caused by *Puccinia recondita* sp. *tritici* was observed in PHR 4819 and PHR 4947 lines, which in 2018 and 2019 were characterized by resistance of 9 on a 9° scale (Table 2). In the case of line PHR 4819, marker *wmc44* of gene *Lr46* was identified during molecular analyses, while markers of both analyzed genes (*Lr34* and *Lr46*) were identified in line PHR 4947. Genotypes in which none of the markers of the analyzed genes were found were characterized by an average field resistance from 3.5 in 2018 (PHR 4859) to 8 in 2017 (PHR 4670, PHR 4922, PHR 4995, and PHR 4997).

**Table 2:** Field and laboratory evaluation of 64 wheat cultivars

Degree	Genotype name	Field infection score		Laboratory evaluation	
		9° scale	*Lr34*	*Lr46*	
		2017	2018	*csLV34*	X*wmc44*
1	PHR 4666	6	4	−	+
2	PHR 4670	8	7	−	−
3	PHR 4671	7	5.3	−	+
4	PHR 4672	9	8.5	−	+
5	PHR 4777	8	7	−	+
6	PHR 4792	5	6	−	+
7	PHR 4794	5	6	−	+
8	PHR 4795	8	7.5	−	+
9	PHR 4796	6	6	−	+
10	PHR 4800	6	8.3	−	−
11	PHR 4813	7	5.5	−	+
12	PHR 4817	6	6	−	+
13	PHR 4818	8	8	−	+
14	PHR 4819	9	9	−	+
15	PHR 4820	9	8.8	−	+
16	PHR 4856	8	8	−	+
17	PHR 4859	7	3.5	−	−
18	PGR 4860	7	3.8	−	+
19	PHR 4861	9	7.8	−	+
20	PHR 4862	8	6.5	−	+
21	PHR 4867	8	5	−	+
22	PHR 4868	9	6	−	+
23	PHR 4870	9	7.5	−	+
24	PHR 4872	9	8.5	−	+
25	PHR 4875	8	8.8	−	+
26	PHR 4876	6	5.5	−	+
27	PHR 4877	9	5.8	−	+
28	PHR 4879	8	7	−	+
29	PHR 4888	8	7	−	+
30	PHR 4895	7	8	−	+
31	PHR 4896	8	8.3	−	+
32	PHR 4897	5	4.8	−	+
33	PHR 4903	8	7	−	+
34	PHR 4905	9	8.3	−	+
35	PHR 4906	8	8	−	+
36	PHR 4907	6	5.2	−	−
37	PHR 4922	8	7.2	−	−
38	PHR 4923	6	6.25	−	+
39	PHR 4932	8	6	−	+
40	PHR 4933	6	6.3	−	+
41	PHR 4934	8	7	−	+
42	PHR 4936	9	8.75	−	+
43	PHR 4937	8	6	−	+
44	PHR 4947	9	9	+	+
45	PHR 4948	7	4.5	−	+
46	PHR 4949	7	6.3	−	−
47	PHR 4953	9	6	−	+
48	PHR 4955	9	8.3	−	+
49	PHR 4956	7	5	−	+
50	PHR 4957	5	7.3	−	−
51	PHR 4962	9	7.8	−	+
52	PHR 4963	7	6.3	−	+
53	PHR 4973	7	5.5	−	+
54	PHR 4977	9	8.7	−	+
55	PHR 4979	7	6	−	+
56	PHR 4995	8	7.3	−	−
57	PHR 4997	8	6	−	−
58	PHR 5000	8	7.5	−	+
59	PHR 5003	6	6.3	−	+
60	PHR 5004	7	5.8	−	+
61	PHR 5005	9	8.8	−	+
62	PHR 5007	9	7.3	−	+
63	PHR 5013	9	8.5	−	+
64	PHR 5017	9	6.8	−	+

+ variety of the analyzed gene.

− variety without the analyzed gene.

## 4 Discussion

Genetic disease resistance is a highly desirable trait in plants [17,18]. It is an environmentally friendly and definitely more profitable alternative to cultivations that use pesticides. The genome of common wheat (*Triticum aestivum* L. ssp*. aestivum*) is characterized by the presence of a number of genes with high resistance potential [12,19,20]. Unfortunately, most of the long-functioning resistance genes are not effective against current breeds of *P. recondita* f. sp. *tritici*, *P. striiformis* f. sp. *tritici*, *P. graminis*, and *Blumeria graminis*, causing leaf rust, stripe rust, stem rust, and powdery mildew, respectively [21]. Leaf rust is a fungal disease that is one of the biggest threats to present common wheat cultivation [22]. Because of the systematic occurrence and significant reduction in wheat yields, leaf rust is a disease of great economic importance and is associated with yield decreases ranging from 25 to even 45% [23].

The study determined the resistance of 64 common wheat lines originating from the experimental station of Poznańska Hodowla Roślin sp. z o.o. in Poland in the years 2017 and 2018, in field conditions. PHR 4819 and PHR 4947 lines proved to be the most resistant. Both lines were characterized by 9° of resistance in both years. Because of the favorable weather conditions during the growing season in 2017, the vast majority of the lines analyzed had a higher degree of resistance compared to 2018. Resistance in 2017 ranged from 5 to 9°, with as many as 34 lines with resistance at the level of 8–9°.

According to Krattinger et al. [24], *Lr* genes present in wheat and other cereals can be divided into three groups, regarding their stability and specificity. The first of these includes the major genes, also called R genes, which confer racial-specific resistance to one race of the pathogen. Most of the identified *Lr* genes belong to this group. Proteins encoded by R genes directly or indirectly receive virulence effects induced by pathogens that are secreted into the cytoplasm of host plant cells. The second group includes genes that are characterized by nonspecific resistance, comprising many races of fungal pathogens. Examples of genes with such resistance are the well-studied genes *Lr34* and *Lr46*. The third group of genes, like the second, includes genes that confer nonspecific resistance; however, resistance is raised against all races within the same pathogen species. A known example of a gene with such resistance is *Yr36*, which provides resistance to stripe rust in wheat [25].

Most of the known *Lr* genes have racial-specific resistance that occurs at the seedling stage. However, there are several genes, including *Lr12*, *Lr13*, *Lr22a*, *Lr34*, *Lr46*, *Lr67*, *Lr68*, and *Lr77*, also called APR genes, which exhibit resistance at the adult plant stage. The latest literature indicates that rust resistance, which results from the combination of genes conferring racial-specific resistance and genes not specific to a given race of the pathogen, is the more persistent and the most preferred resistance in cultivated wheat cultivars [26]. In common wheat, rust resistance genes from related wild forms of *Agropyron*, *Aegilops*, and *Scale* are currently applied [27].

To date, at least four effective gene complexes have been identified: *Lr34/Yr18/Sr57/Pm38*, *Lr46/Yr29/Pm39*, *Lr67/Yr46*, and *Sr2/Yr30*, which provide partial resistance but last for a long period. This type of resistance is referred to as slow rust and causes slower disease development [21]. Gene *Lr34* codes ATP-binding transporter (ABC transporter – ATP-binding cassette transporter) [24]. The gene *Lr34* is present in European cultivars that have Mentana and Ardito cultivars in their pedigree registered in the early twentieth century [28]. Frontana is one of these cultivars.

In the current study, Frontana was used as a reference variety in which the presence of marker *csLV* of gene *Lr34* and marker *wmc44* of gene *Lr46* was confirmed. The *Lr34* gene marker was developed by Lagudah et al. [29]. SSR-PCR marker *csLV* was identified during analysis in line PHR 4947. The presence of a specific sequence has also been confirmed in multiplex PCR analyses. In addition to gene *Lr34*, gene *Lr46* was identified in this genotype. Lines PHR 4947 and PHR 4819 were characterized by the highest leaf rust resistance in field conditions. For years, the authors have been analyzing the genotypes of various species for the presence of *Lr34* gene.

Singh and Huerta-Espino [30] studied a doubled haploid population for the presence of the gene *Lr34* marker. Wheat studied by the authors was created from a cross between Japanese Fukuho-komugi wheat and Israeli wheat oligoculm. In another experiment, Fukuho-komugi showed leaf necrosis in field trials in Mexico and was therefore found to be a carrier of the *Lr34*/*Yr18* gene complex. In addition, the latency period of the disease increased, and the size and openness of uredinium decreased. Although the effects of gene *Lr34* expression could be observed at every stage of leaf growth, the differences in the latent period and in the number of open uredinia increased significantly, starting from phases 4 to 5. The relative effect of gene *Lr34* expression on the latent period was reduced, and with increasing temperature, its sensitivity was also elevated. Temperature and growth stage had the lowest impact on the size of uredinium [30].

Reynolds and Borlaug [23] conducted research on lines with and without the gene *Lr34*. The authors showed that thecrop loss in cultivars with *Lr34* ranged from 11 to 15%, whereas in the absence of this gene, losses ranged from 40 to even 85%, depending on the sowing date.

The gene *Lr34* is effective at the adult plant stage and, under favorable conditions, i.e., 4–10°C, at the flag leaf stage and seedling stage [31]. Unfortunately, the resistance conditioned by *Lr34* is less effective at high temperatures [32]. Inoculation tests performed in field conditions on inbred wheat lines (F6) with the *Lr34res* allele showed 50% infestation with a 15% reduction in yield compared to control forms, in which 100% infestation and a 84% decrease in yield were observed [7].

The second of the analyzed genes was *Lr46*, which is also present as a gene complex and is linked to gene *Yr29*, which shows the resistance capacity to stripe rust. The gene *Lr46* has been identified in the variety “Pavon 76” on chromosome 1B [33]. Its sequence is still unknown, but a number of candidate genes have already been selected.

During our own STS-PCR analyses, the marker *wmc44* of gene *Lr46* was identified in most of the analyzed lines. This marker was not present in the following genotypes: PHR 4670, PHR 4800, PHR 4859, PHR 4907, PHR 4922, PHR 4949, PHR 4957, PHR 4995, and PHR 4997. The presence of a specific sequence has also been confirmed in multiplex PCR analyses. In addition to the specific 242 bp product characteristic for *Lr46*, nonspecific products appeared. Genotypes carrying markers of the analyzed genes were characterized by good resistance to leaf rust in the field in both 2018 and 2019. In 2018, the resistance ranged from 5 to 8° and in 2019 from 5.2 to 8.3°, except for line 4859 where 3.5° was recorded.

This gene has been the research object of many scientists. Singh et al. [33] investigated the genetic associations of *Lr46* with leaf rust resistance of adult plants. The authors conducted their works on two cultivars “Pavon 76” and “Avocet S,” which were analyzed using the AFLP-PCR technique. Preliminary studies have shown that inbred lines of wheat carrying this gene were characterized by an average of 35% lower infestation (leaf rust) compared to control forms in an experiment where plants were inoculated with fungal spores. These studies also allowed us to develop an STS (sequence-tagged site) marker *wmc44*, linked to the *Lr46* gene locus [11]. In turn, Agarwal and Saini [9] proved that gene *Lr46* was ineffective against leaf rust races in India. Lack of expression of this gene in some regions is explained by the temperature that is optimal for the development of the pathogen and unfavorable for the resistance mechanism conditioned by this gene [34]. Other traits of partial resistance of adult plants that exert the slow rust effect have been compared between *Lr34* and *Lr46* by Martinez et al. [8].

## 5 Conclusions

In recent years, molecular techniques have become the main tool used in breeding and protecting crop plants. They are used in genotype identification, genetic diversity studies, and genetic map construction. Genetic markers efficiently support the effectiveness of traditional methods based on morphological and phenotypic analyses, which require considerable time and labor. MAS selection using multiplex PCR contributes to the rapid identification of cultivars showing desired functional characteristics, which makes resistance breeding possible. Research shows that the molecular markers *csLV34* and X*wmc44* enable the identification of genes *Lr34* and *Lr46*. It is possible to use the multiplex PCR technique for simultaneous identification of both genes, which will shorten the time of selection. PHR line 4947 is a genotype that can be successfully used for further crossbreeding as the source of genes *Lr34* and *Lr46*. This line is also characterized by high resistance in field conditions (9°).
